# Public perspectives on availability, accessibility and quality of midwifery services in the United Arab Emirates (UAE): Thematic analysis of an open-ended survey question

**DOI:** 10.18332/ejm/219214

**Published:** 2026-07-03

**Authors:** Maeve Anne O'Connell, Khadija Al Sulaimi, Rasha Salah Eweida, Joanna Andrews, Joeri Vermeulen

**Affiliations:** 1 Midwifery Department, Fatima College of Health Sciences, Abu Dhabi, United Arab Emirates; 2 School of Nursing and Midwifery, Brookfield Health Sciences Complex, University College Cork, Cork, Ireland; 3 Latifa Hospital, Dubai, United Arab Emirates; 4 Nursing Department, College of Health and Sport Sciences, University of Bahrain, Zallaq, Bahrain; 5 Psychiatric and Mental Health Nursing Department, Faculty of Nursing, Alexandria University, Alexandria, Egypt; 6 Belgium Department of Life Sciences and Medicine, University of Luxembourg, Eschsur-Alzette, Luxembourg; 7 Department of Public Health, Biostatistics and Medical Informatics Research Group, Vrije Universiteit Brussel, Brussels, Belgium

**Keywords:** public health, midwifery, midwifery workforce, service accessibility, maternal health services

## Abstract

**INTRODUCTION:**

The UAE is facing a critical shortage in the midwifery workforce. Maternity care is predominantly obstetric-led. The UAE government is committed to increasing the number of midwives by 38 per year until 2030 and has invested in midwifery education, with the first direct-entry Bachelor’s degree programs in midwifery available. However, little is known about public perspectives on the availability, accessibility and quality of midwifery services in the UAE. Our aim was to explore public perspectives on challenges and gaps in midwifery services in the UAE.

**METHODS:**

An exploratory qualitative study was conducted using responses to an open-ended question within a bilingual (English/Arabic) online survey distributed across the UAE via social media and snowball sampling from November 2024 to February 2025. Responses to the open-ended question were analyzed thematically.

**RESULTS:**

Out of 207 (100%) survey respondents, 119 (57%) answered the open-ended question. Thematic analysis identified five central themes: limited availability and accessibility of midwifery services; cultural and public perceptions of midwives; limited scope of practice and autonomy for midwives; education, awareness and professional development; and quality and scope of postpartum care.

**CONCLUSIONS:**

The findings highlight significant public support for the expansion and enhancement of midwifery services in the UAE. Tackling the identified challenges has the potential to improve the quality and accessibility of maternity care, empower women’s choices and strengthen midwives' contribution to national maternal health objectives. Embedding midwifery reforms into national strategies will help the UAE meet its healthcare goals, including those outlined in Vision 2030, by building a sustainable, evidence-based, high-quality maternity care system.

## INTRODUCTION

Midwifery is proposed as a global solution for Universal Health Coverage^1^ and meeting the Sustainable Development Goals (SDGs), a global policy framework that includes targets to reduce maternal and neonatal mortality and improve access to essential health services^2,3^. When educated to international standards and supported by a well-functioning health system and enabling environment, midwives can deliver up to 87% of the essential care required for women and newborns^2^. Furthermore, investment in midwifery is recommended to reduce maternal and neonatal mortality and strengthen the availability of maternal and newborn health services^1^. Midwifery models of care promote a respectful and collaborative approach to maternity care^2,4^. Health workers lacking the full range of midwifery competencies are unable to deliver the same quality of care or achieve the life-saving outcomes that internationally qualified midwives can provide^1^.

In the United Arab Emirates (UAE), midwives represent a small proportion of the maternal healthcare workforce. Recent WHO workforce estimates indicate a continued imbalance between nurses and midwives nationally, reflecting ongoing reliance on a predominantly nursing-led maternity workforce. In response, strategic efforts are underway to expand the midwifery workforce through national workforce planning, expansion of midwifery education pathways and strengthening of regulatory and professional frameworks aligned with international standards^5^. Addressing this gap also requires sustained investment in high-quality midwifery education to support a skilled and sustainable workforce^6,7^. Despite these developments, public awareness of midwives’ scope of practice, professional autonomy and role across the continuum of maternity care remains limited. In this study, the term ‘public’ refers to adults residing in the UAE recruited through online platforms and social networks, including individuals with and without direct experience of pregnancy or maternity services. Public perception of healthcare professionals is an important determinant of service utilization, trust in care providers and policy acceptability. Evidence suggests that public understanding of professional roles influences patterns of help-seeking, care pathways and engagement with available services^8,9^. This is particularly evident in maternity care, where choice of provider is shaped by knowledge, cultural expectations and perceived safety^10,11^. In settings where midwifery is less visible within the health system, limited awareness may constrain appropriate utilization of midwifery-led services and hinder implementation of evidence-based models of care. Understanding public perceptions is therefore essential to inform workforce planning, public education strategies and health system reforms aimed at strengthening midwifery integration^3,12,13^. A 2014 survey of pregnant women in the UAE (n=496) revealed that one-third of respondents lacked knowledge or understanding of the midwife’s role and one in ten believed there were no midwives practicing in Abu Dhabi^6^. These findings highlight the pressing need to both strengthen midwifery services and improve public understanding of their value.

Public perception plays a vital role in shaping health policy, service use and workforce planning^9^. Understanding how communities view the availability, accessibility and quality of midwifery services is essential to ensuring that care is trusted and delivered effectively.

Despite growing recognition of midwives’ impact, limited data exist, particularly in the UAE, on public understanding of their role. Without this insight, efforts to expand midwifery care may face barriers. Public perception in this context refers to people’s expectations, experiences and perceived needs regarding midwifery services. Gathering public perspectives is therefore critical to developing responsive, culturally appropriate services^10^.

Accordingly, this study aimed to explore public perspectives on the challenges and gaps in the availability, accessibility and quality of midwifery services in the UAE.

## METHODS

### Study design

This study employed a cross-sectional survey design incorporating both quantitative items and a qualitative open-ended question. Quantitative survey items assessed awareness, knowledge and satisfaction with midwifery services, while a single open-ended question explored perceived challenges and barriers to accessing midwifery care^14^. The qualitative component enabled deeper exploration of participants’ perceptions of midwifery roles, experiences with maternity services and perceived barriers to care. Open-text responses were analyzed using reflexive thematic analysis as described by Braun and Clarke^15^. The broader survey findings have been reported previously in relation to public knowledge of midwifery roles in the UAE^8^. The present work reports the qualitative analysis of responses to a single open-ended question included in that survey.

### Setting

The study was conducted in the UAE from November 2024 to February 2025. Maternity care in the United Arab Emirates (UAE) is predominantly hospital-based and largely obstetric-led across both public and private sectors. Although midwives are employed within maternity services, models of care remain primarily physician-led and midwifery-led continuity models are not widely implemented. Most women receive antenatal and intrapartum care within obstetric services, with midwives often working in supportive clinical roles rather than as lead care providers. Care pathways vary by emirate and facility but typically include consultant-led and shared-care models, with limited access to community-based or midwifery-led services. Understanding this service configuration is essential for interpreting patterns of public awareness and engagement with midwifery care.

### Participants

Participants were adults aged ≥18 years residing in the United Arab Emirates (UAE), recruited from the general population via online platforms and social media networks. Individuals were eligible if they lived in the UAE and could complete the bilingual (English/Arabic) survey. No exclusion criteria were applied beyond age and residency requirements. The sample included individuals with and without direct experience of pregnancy or maternity services, reflecting the study’s aim to assess broader public awareness alongside experiential perspectives. Sociodemographic variables (including age, gender, nationality, education level and prior maternity care experience) were collected to contextualize findings and explore variation across population groups.

### Data collection

An anonymous survey was widely shared online via social media accounts, LinkedIn, paid Instagram ads and snowball sampling of acquaintances. The main survey consisted of the Midwife Profiling Survey^14^ and was available in English and Arabic. The midwife profiling survey consists of 10 questions, which are designed to assess knowledge about midwifery competencies and has been validated in English^14^.

This study presents findings of the responses to the following open-ended question: ‘From your perspective, what challenges or gaps do you see in the availability, accessibility, or quality of midwifery services in the UAE?’. Please share any specific areas where you feel improvements or additional support are needed.

### Data analysis

The Arabic surveys were translated into English and the analysis was conducted in English. Reflexive thematic analysis, following Braun and Clarke’s six-phase approach, was conducted^15^. The whole dataset was examined to familiarize researchers with the data through repeated reading and reflection. After familiarization with the data, initial codes were generated. In the third phase, two researchers (JV and MOC) independently grouped the codes into recurrent patterns and candidate themes. In the fourth phase, the researchers met to discuss and agree on the subthemes and broader themes to ensure clarity and close reflection of the data. In the final phase, the themes were reviewed against the dataset and overlap was minimized to ensure that the analysis remained grounded in participants’ accounts.

## RESULTS

Table 1 presents the themes and subthemes identified through the thematic analysis. The final sample included 119 responses to the open-ended question. Most respondents were female (85.7%), aged 26–39 years (41.2%) and married (70.5%). Emiratis accounted for 19.3% of the sample and 34.3% had completed postgraduate education. The majority (73.9%) were from non-healthcare backgrounds, while 26.1% were healthcare professionals.

**Table 1:** Final themes and subthemes identified from thematic analysis of an open-ended survey question on midwifery services in the United Arab Emirates, November 2024 to February 2025 (N=119)

*Themes*	*Limited availability and accessibility of midwifery services*	*Cultural and public perception of midwives*	*Limited scope of practice and autonomy for midwives*	*Education, awareness, and professional development*	*Quality and scope of postpartum care*
**Subthemes**	Insufficient midwife numbers Lack of home visits Limited midwife-led birth centers	Lack of awareness Preference for obstetricians Language and cultural barriers	Lack of autonomy in practice Hospital-dependent roles Regulatory barriers	Need for education and awareness Lack of specialized training Continuous professional development for midwives	Limited postpartum support Lack of postpartum home care Inadequate breastfeeding support

Five themes were identified: limited availability and accessibility of midwifery services; cultural and public perceptions of midwives; limited scope of practice and autonomy for midwives; education, awareness and professional development; and quality and scope of postpartum care.

### Limited availability and accessibility of midwifery services

Participants frequently described limited availability of midwifery services in the UAE, often attributing this to workforce shortages and restricted service provision across settings. Responses suggested that access to midwifery care was perceived as uneven, with participants noting limited visibility of midwives within maternity services and restricted availability through insurance coverage. These perceptions also reflected broader concerns about the predominance of hospital-based, consultant-led care models, which were viewed as limiting opportunities for midwifery-led pathways. As one participant noted:

*‘Their number is small.’* (Arabic-speaking participant, Emirati woman, aged 18–25)

Participants additionally highlighted gaps in service accessibility across the continuum of care, particularly the limited availability of postnatal home visiting and midwifery-led birth centers. These services were viewed as important for improving continuity, choice and person-centered care, particularly for low-risk pregnancies. As one respondent explained:

*‘We would be interested in birth centers run by midwives.’* (English-speaking participant, American woman, aged 26–39)

### Cultural and public perceptions of midwives

Participants frequently described limited public awareness of midwifery roles within the UAE, often expressing uncertainty about midwives’ involvement across pregnancy, birth and the postnatal period. These responses suggest that midwifery remains relatively unfamiliar within the broader cultural context, contributing to the underutilization of midwifery services and a continued preference for obstetric-led care. Several participants highlighted that maternity care decisions were strongly shaped by cultural expectations and perceptions of medical authority. As one participant noted:

*‘The idea of midwifery in the UAE is not that established yet, and so traditionally they would look for doctors more and would not see the importance of midwives.’* (English-speaking participant, Filipina woman, aged 18–25)

Gender differences in awareness were also evident, with male participants more likely to report uncertainty about midwifery roles. While most expressed limited knowledge, a small number demonstrated supportive attitudes towards expanding midwifery services. Participants further identified structural and cultural barriers affecting access, including language differences, perceived shortages of Arabic-speaking midwives and geographical variation in service availability. As one respondent explained:

*‘There are not many Arab midwives in the UAE, this creates a language barrier for non-English speaking women as they have the right to feel understood and supported by their midwives.’* (Emirati woman, aged 18–25)

### Limited scope of practice and autonomy for midwives

Participants frequently perceived midwives in the UAE as having limited professional autonomy, often describing their roles as constrained by organizational structures and physician-led models of care. Responses suggested that midwives were commonly viewed as working within restricted clinical boundaries, with limited opportunities to practice independently across the continuum of maternity care. Participants also linked these constraints to hospital-based employment models and broader system-level factors, including regulatory frameworks and the dominance of obstetric-led services. As one participant explained:

*‘Midwives in the UAE should be empowered and granted full authority, including the ability to prescribe medication, as in other countries.’*

Some respondents further highlighted the limited visibility of midwifery-led care pathways, noting that midwives were often perceived as subordinate to physicians rather than autonomous practitioners. As another participant stated:

*‘Giving midwives a greater opportunity to practice their specialty, meaning they can manage a clinic, for example and refer cases that need doctors.’* (Arabic-speaking, Emirati woman, aged 18–25)

### Education, awareness and professional development

Participants frequently emphasized the importance of strengthening both public awareness and professional development in midwifery. Many responses reflected perceived gaps in understanding of midwives’ roles among the public, alongside concerns about variation in professional preparation and ongoing training. These views suggest that awareness of midwifery in the UAE remains closely linked to perceptions of professional expertise and service quality. Some participants specifically identified breastfeeding support as an area where additional specialist training was needed, indicating expectations for midwives to provide comprehensive postnatal care. As one participant noted:

*‘There should be a focus on educating people about the role of midwives and their importance.’* (Arabic-speaking participant, aged 26–39)

Participants also highlighted the perceived need for ongoing professional development to support consistent, high-quality care across maternity services.

### Quality and scope of postpartum care

Participants frequently identified gaps in postpartum care provision, particularly in relation to continuity of support following hospital discharge. Many described postnatal services as limited in scope and duration, with concerns raised about insufficient specialist support for maternal recovery, breastfeeding and emotional well-being. These accounts suggest that postpartum care is perceived as less prioritized within maternity services, with ongoing reliance on hospital-based care models and limited access to community-based midwifery support. Some participants attributed these gaps to the predominant role of nurses in postnatal wards, perceiving variability in expertise related to postnatal recovery and breastfeeding support. As one participant explained:

*‘Postpartum care is limited and nurses often don’t fully understand the needs of mothers who have just given birth.’* (English Arabic-speaking participant, UK woman, aged 26–39)

Participants also highlighted the absence of structured community follow-up, particularly home visiting services, which were viewed as important for supporting maternal adjustment and infant care in the early postnatal period. As one respondent noted:

*‘Midwives should visit mothers at home to provide postpartum support.’* (English-speaking participant, UK woman, aged 26–39)

## DISCUSSION

Midwifery services in the UAE remain constrained by limited accessibility, availability and professional autonomy. The findings of this study highlight persistent structural and organizational barriers that restrict midwives’ ability to practice to the full scope of their education and training. Despite the recognized potential of midwives to deliver high-quality, woman-centered care, participants described maternity services as predominantly hospital-based and obstetric-led, with limited visibility of midwifery-led pathways. These findings align with international evidence demonstrating that midwives are most effective when supported by enabling environments that promote autonomy, continuity of care and integration within health systems^14-16^.

Structural barriers were consistently identified across themes, including limited availability of midwifery-led services, restricted access to community-based care and perceived gaps in continuity across the maternity continuum. Participants highlighted the absence of midwife-led birth centers, limited provision of postnatal home visiting and restricted opportunities for midwives to function as primary care providers. These findings reflect broader system-level challenges identified globally, where inadequate integration of midwives into service delivery limits access to appropriate care and constrains workforce effectiveness^14,16^. Addressing these gaps through service redesign and stronger integration of midwifery within primary healthcare systems may improve access, continuity and person-centered care in the UAE context.

A key finding of this study was the perceived inadequacy of postpartum support, particularly within the community setting. While intrapartum care often receives greater policy and clinical attention, the postnatal period is critical for maternal recovery, infant health and family wellbeing. Participants described gaps in breastfeeding support, continuity of care and psychosocial support, consistent with international evidence linking inadequate postnatal services to reduced breastfeeding duration and poorer maternal outcomes^17^. In high-performing health systems, midwives commonly contribute to postnatal home visiting, breastfeeding support and early parenting guidance. The absence of similar models in the UAE represents a missed opportunity to strengthen continuity and optimize maternal and newborn outcomes. Ensuring the quality of midwifery care also requires sustained investment in continuing professional development (CPD). Participants’ perceptions of variation in expertise highlight the importance of structured opportunities for midwives to maintain and advance competencies across the continuum of maternity care, including breastfeeding support, postnatal care and evidence-based clinical practice. International evidence demonstrates that ongoing education and supportive professional environments are central to maintaining safe, effective and person-centered maternity services. Strengthening CPD frameworks in the UAE may therefore enhance consistency in care provision, support professional confidence and increase public trust in midwifery services.

Public awareness and cultural perceptions also emerged as important influences on service utilization. Participants frequently described uncertainty about midwives’ roles and expressed strong cultural preferences for obstetric-led care. These findings suggest that limited visibility of midwives within maternity services contributes to underutilization and reinforces existing care-seeking patterns. Improving public understanding of midwifery through targeted education initiatives may support more informed engagement with available care options and strengthen the implementation of midwifery-led models.

From a health systems perspective, these findings highlight the need for sustained investment in the midwifery workforce, including scaling up education, strengthening regulatory frameworks and supporting enabling practice environments. Such reforms align with WHO recommendations to strengthen primary healthcare and reduce maternal and newborn morbidity and mortality through optimized midwifery care^16^. From a rights-based perspective, improving access to midwife-led services also supports women’s autonomy, choice and equitable access to respectful maternity care^18^. Aligning these reforms with national strategies, including the UAE Vision 2030 agenda, may further support the integration of midwifery within broader health system strengthening efforts^19,21^.

### Strengths and limitations

A strength of this study was its wide reach through social media, enabling diverse perspectives and allowing participants to voice their experiences through an open-ended question. This enhanced the richness and relevance of the data. Limitations include the potential for self-selection bias, as participation was limited to those with internet access and interest in the topic, and the inability to probe responses in greater depth, as might be possible in interviews or focus groups. Although the findings are context-specific to the UAE, the issues identified, such as access, availability and postpartum support, mirror global challenges in midwifery.

## CONCLUSIONS

This study highlights significant gaps in the accessibility, availability and autonomy of midwifery services in the UAE, particularly in community-based postpartum support and breastfeeding care. Strengthening midwifery education, expanding the scope of practice and integrating midwives into primary and community healthcare are critical steps toward improving maternal and newborn outcomes. Aligning these reforms with the UAE’s Vision 2030 and national maternal and child health strategies would enable midwives to practice to their full potential, advancing quality, equity and sustainability in healthcare while improving outcomes for women and families.

## Data Availability

The datasets generated and analyzed during the current study are not publicly available due to the qualitative nature of the data and the potential for identification of participants. De-identified data may be available from the corresponding author on reasonable request, subject to institutional ethical approval.

## References

[1] Renfrew MJ, Malata AM. Scaling up care by midwives must now be a global priority. Lancet Glob Health. 2021;9(1):e2-e3. doi:10.1016/S2214-109X(20)30478-2 33275951

[2] Nove A, Friberg IK, de Bernis L, et al. Potential impact of midwives in preventing and reducing maternal and neonatal mortality and stillbirths: a lives saved tool modelling study. Lancet Glob Health. 2021;9(1):e24-e32. doi:10.1016/S2214-109X(20)30397-1 33275948 PMC7758876

[3] Renfrew MJ, McFadden A, Bastos MH, et al. Midwifery and quality care: findings from a new evidence-informed framework for maternal and newborn care. Lancet. 2014;384(9948):1129-1145. doi:10.1016/S0140-6736(14)60789-3 24965816

[4] Nove A, Boyce M, Neal S, et al. Increasing the number of midwives is necessary but not sufficient: using global data to support the case for investment in both midwife availability and the enabling work environment in low- and middle-income countries. Hum Resour Health. 2024;22(1):54. doi:10.1186/s12960-024-00925-w 39039518 PMC11264417

[5] Abu Dhabi Health Workforce Plan. Department of Health (DoH); 2020. Accessed March 16, 2026. https://www.doh.gov.ae/en/investor/abu-dhabi-health-workforce

[6] Edwards G, Elsori D, Sarr M. What is a midwife? A survey of pregnant women in Abu Dhabi. Pract Midwife. 2014;17(6):31–34. Accessed March 16, 2026. https://pubmed.ncbi.nlm.nih.gov/25004702/25004702

[7] O’Connell MA, Sosa G. Midwifery in Abu Dhabi: a descriptive survey of midwives. Women Birth. 2023;36(4):e439-e444. doi:10.1016/j.wombi.2023.02.002 36822961

[8] Levesque JF, Harris MF, Russell G. Patient-centred access to health care: conceptualising access at the interface of health systems and populations. Int J Equity Health. 2013;12:18. doi:10.1186/1475-9276-12-18 23496984 PMC3610159

[9] Babitsch B, Gohl D, von Lengerke T. Re-revisiting Andersen’s behavioral model of health services use: a systematic review of studies from 1998-2011. Psychosoc Med. 2012;9:Doc11. doi:10.3205/psm000089 23133505 PMC3488807

[10] Sandall J, Soltani H, Gates S, Shennan A, Devane D. Midwife-led continuity models versus other models of care for childbearing women. Cochrane Database Syst Rev. 2016;4(4):CD004667. doi:10.1002/14651858.CD004667.pub5 27121907 PMC8663203

[11] Downe S, Finlayson K, Oladapo OT, Bonet M, Gülmezoglu AM. What matters to women during childbirth: a systematic qualitative review. PLoS One. 2018;13(4):e0194906. doi:10.1371/journal.pone.0194906 29664907 PMC5903648

[12] ten Hoope-Bender P, de Bernis L, Campbell J, et al. Improvement of maternal and newborn health through midwifery. Lancet. 2014;384(9949):1226-1235. doi:10.1016/S0140-6736(14)60930-2 24965818

[13] Vedam S, Stoll K, Taiwo TK, et al. The giving voice to mothers study: inequity and mistreatment during pregnancy and childbirth in the United States. Reprod Health. 2019;16(1):77. doi:10.1186/s12978-019-0729-2 31182118 PMC6558766

[14] Vermeulen J, Peersman W, Quadvlieg L, et al. Development and validation of the midwife profiling questionnaire assessing women’s preferred perinatal care professional and knowledge of midwives’ legal competences. Sex Reprod Healthc. 2018;16:23-32. doi:10.1016/j.srhc.2018.01.003 29804771

[15] Braun V, Clarke V. Using thematic analysis in psychology. Qual Res Psychol. 2006;3(2):77–101. doi:10.1191/1478088706qp063oa

[16] The State of the World’s Midwifery 2021. United Nations Population Fund; May, 2021. Accessed March 16, 2026. https://www.unfpa.org/publications/sowmy-2021

[17] Victora CG, Bahl R, Barros AJ, et al. Breastfeeding in the 21st century: epidemiology, mechanisms and lifelong effect. Lancet. 2016;387(10017):475-490. doi:10.1016/S0140-6736(15)01024-7 26869575

[18] Respectful maternity care: the universal rights of mothers and newborns. Healthy Newborn Network; 2019. Accessed March 16, 2026. https://www.healthynewbornnetwork.org/hnn-content/uploads/Respectful-Maternity-Care-Charter-2019.pdf

[19] Supreme Council for Motherhood and Childhood. National Strategy for Motherhood and Childhood 2017–2021. In Arabic. United Arab Emirates: Supreme Council for Motherhood and Childhood; 2017. Accessed June 25, 2026. https://assets.u.ae/api/public/content/80f7e757b21d4b77beb694fb8f148abf?v=c9f89c57

[20] Abu Dhabi Economic Vision 2030. Abu Dhabi Centre for Technical and Vocational Education and Training; 2007. Accessed March 16, 2026. https://www.actvet.gov.ae/en/media/lists/elibraryld/economic-vision-2030-full-versionen.pdf

[21] UAE National Strategy for Nursing/Midwifery: A Roadmap to 2026. United Arab Emirates Ministry of Health and Prevention; 2022. Accessed June 25, 2026. https://assets.u.ae/api/public/content/a37e48089eb841d8ba0b7b905ba390a6?v=b3985765 or https://uaesupremecouncil.org/en/about-the-uae/strategies-initiatives-and-awards/strategies-plans-and-visions/health/national-strategy-for-nursing-and-midwifery.html

